# Role of mitochondrial dysfunction in ocular surface diseases

**DOI:** 10.15698/cst2025.08.309

**Published:** 2025-08-05

**Authors:** Xiaohan Chen, Jiaxu Hong, Qihua Le

**Affiliations:** 1 Department of Ophthalmology, Eye & ENT Hospital, Fudan University, Shanghai 200031, China.; 2 Department of Ophthalmology, Eye & ENT Hospital, State Key Laboratory of Medical Neurobiology and MOE Frontiers Center for Brain Science, Fudan University, Shanghai 200031, China.; 3 Shanghai Key Laboratory of Rare Disease Gene Editing and Cell Therapy; Shanghai Engineering Research Center of Synthetic Immunology, Shanghai, 200032, China.; 4 NHC Key laboratory of Myopia and Related Eye Diseases, Shanghai, 200031, China.; 5 Department of Ophthalmology, Children’s Hospital of Fudan University, National Pediatric Medical Center of China, Shanghai, China.

**Keywords:** mitochondrial dysfunction, the ocular surface diseases, dry eye, Fuchs endothelial cell dystrophy

## Abstract

The dysfunction of mitochondria, the “energy factories” of cells, not only causes an insufficiency of energy production but also leads to various pathological alterations in cells such as the accumulation of reactive oxygen species, inflammatory responses and mitochondrial DNA damage, all of which were involved in the onset or deterioration of diseases. The presence of mitochondrial dysfunction has been confirmed in many ocular surface diseases such as dry eye, Fuchs corneal endothelial dystrophy and diabetic keratopathy. However, its role in the pathogenesis of ocular surface diseases and underlying molecular mechanisms have not been fully elucidated. Moreover, mitochondrial therapies for ocular surface diseases are currently still under investigation. This mini-review summarizes the pathological features of mitochondrial dysfunction and its mechanisms that have been identified in the pathogenesis of ocular surface diseases, and discusses the potential of mitochondrial therapies in the treatment of these diseases.

## Abbreviations

ATP - adenosine triphosphate,

CEnCs - Corneal endothelial cells,

DE - Dry Eyes,

ER - endoplasmic recticulum,

FECD - Fuchs endothelial cell dystrophy,

MAM - mitochondria-associated membranes,

mPTP - mitochondrial permeability transition pore,

mtDNA - mitochondrial DNA,

OXPHOS - oxidative phosphorylation,

ROS - reactive oxygen species.

## INTRODUCTION

Mitochondria, important organelles within eukaryotic cells, are responsible for energy production for cells. Glucose, fatty acids, or amino acids are converted into adenosine triphosphate (ATP) through oxidative phosphorylation (OXPHOS) in mitochondria 1,2. They are also involved in various physiological activities, including β-oxidation of fatty acids 3,4, metabolism of amino acids 5, synthesis of corticosteroid, sex hormones and insulin 6, and cell signaling and programmed cell death (apoptosis) 7.

Mitochondrial dysfunction, which was firstly used in bioenergetics to refer to a series of physiopathological alterations caused by compromised or loss of mitochondrial function 6, usually leads to the impairment of OXPHOS and ATP production and causes an elevated level of reactive oxygen species (ROS) and inflammation 8. Mitochondrial dysfunction has been confirmed to be involved in the pathogenesis of many diseases. In recent years, an increasing number of studies have been published focusing on its role in ocular diseases, among which novel targets for therapies might be explored. Aiming to provide insights into the research of ocular surface diseases, we made a review of the investigations of mitochondrial dysfunction in this realm and discussed its role in the pathogenesis so as to explore potential novel therapeutic targets based on current findings.

## DEFINITION OF MITOCHONDRIAL DYSFUNCTION

Although the initial definition of mitochondrial dysfunction was relatively narrow, it has been expanded to various pathological alterations of mitochondria in responses to environmental triggers or changes, among which mitophagy and mitochondria- associated endoplasmic reticulum (ER) membranes have been highlighted in recent years.

Mitochondria-specific macroautophagy, also called mitophagy, is crucial for the elimination of damaged or dysfunctional organelles and macromolecules through the process of autophagosome formation, lysosome-autophagosome fusion and enzymatic digestion 9,10. Recently, transient receptor potential mucolipin1, a novel channel protein, has been reported to be involved in the clearance of damaged mitochondrial membrane segments by adjacent lysosomes 11.

Mitochondria-associated membranes (MAMs), physically formed by the connection of ER and mitochondria, provide a fundamental platform that allows rapid exchange of biological molecules and maintain cellular health by regulating important cell activities including calcium homeostasis, autophagy, lipid metabolism and cell apoptosis 12,13,14. When stimuli occur, NOD-like receptor protein-3 (NLRP3), a key regulator of inflammation in the cytoplasm, is capable of interacting with MAMs, promoting the assembly and maturation of pro-inflammatory cytokine IL-1ß, accumulating a higher level of oxidative stress in cells and local tissues and consequently leading to inflammation 15,16. There is an increasing number of evidences confirming the involvement of MAMs and NLRP3 in the onset and/or deterioration of inflammatory diseases 17,18,19.

## PATHOLOGICAL FEATURES OF MITOCHONDRIAL DYSFUNCTION

**Figure 1:**
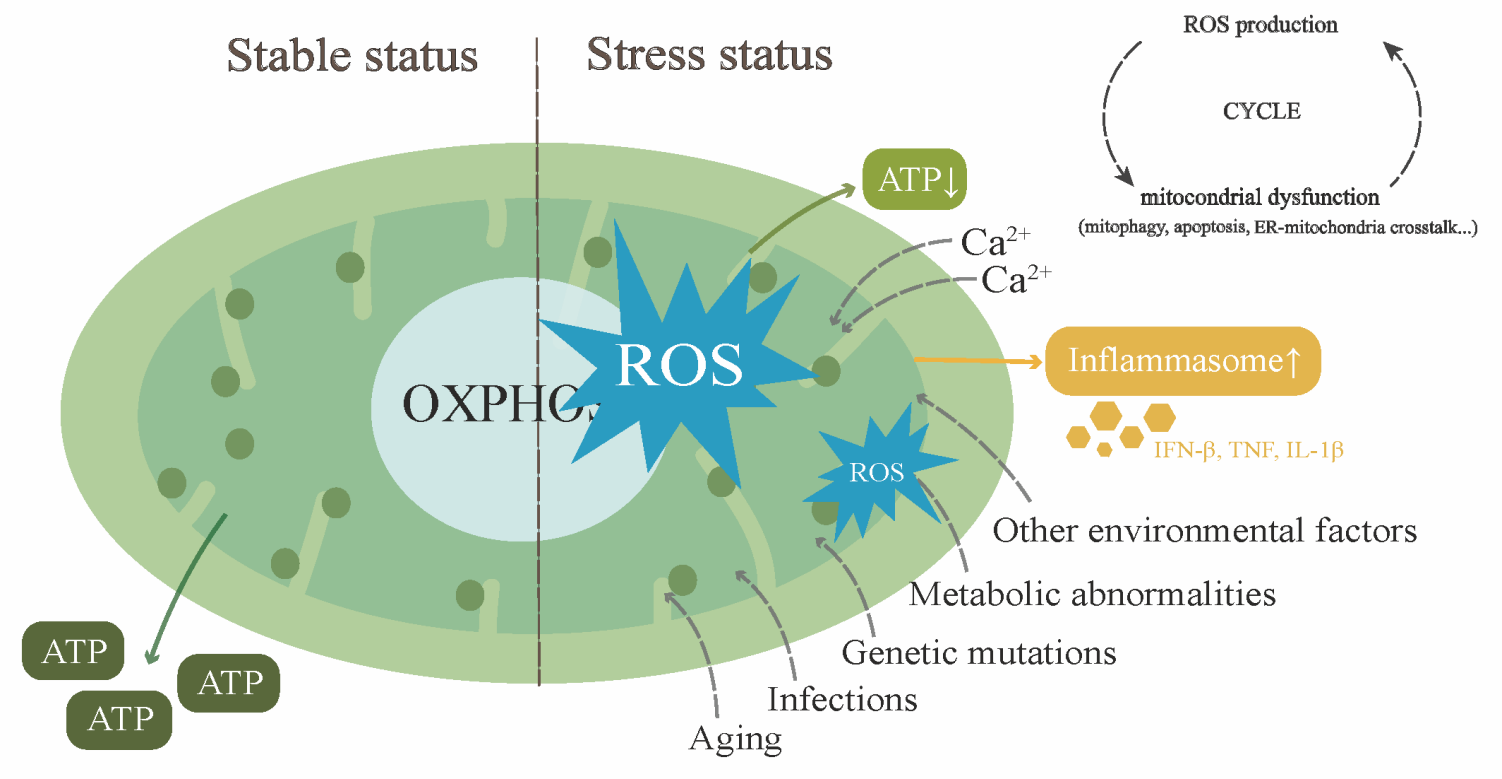
FIGURE 1: The diagram of mitochondria under stable status and stress status. Under stable status, mitochondria are responsible for the production of ATP through oxidative phosphorylation (OXPHOS) in respiratory chain. Under stress from environmental factors, such as aging, gene mutation, infections and metabolic abnormalities, mitochondria undergo oxidative stress, produce large amounts of ROSs and inflammasomes and lead to reduced production of ATP. With the accumulation of ROS, inflammasomes and proinflammatory cytokines, more mitochondria are damaged, causing more severe mitochondrial responses and forming a vicious cycle. Moreover, excessive ROSs could damage mtDNA, leading to mtDNA mutation and impairing the translation and synthesis of key mitochondrial proteins in OXPHOS. (Green dots: ribosomes; ER: endoplasmic reticulum).

The causes for mitochondrial dysfunction are various, including genetic mutations, infections, environmental factors, aging, and metabolic abnormalities (**Figure 1**). They can damage the integrity of the mitochondrial membrane, cause abnormal opening of the mitochondrial permeability transition pore (mPTP), and lead to overload of calcium influx and a reduction of membrane potential, all of which ultimately impair normal function of mitochondria and lead to cellular energy deficiency 1,20,21. When the supply of energy is insufficient, normal physiological activities of cells are severely affected, triggering a wide range of stress responses. Moreover, quality-control proteases 22,23 in mitochondria are activated to remove damaged proteins and transcriptionally induce the expression of chaperones to respond to the stress caused by abnormally folded proteins 24.

The pathological features of mitochondrial dysfunction include membrane depolarization, ROS imbalance and accumulation, mitochondrial DNA (mtDNA) damage and reduced synthesis of ATP 1, covering almost all the mechanisms that are involved in cellular physiological activities. An elevated level of oxidative stress causes the accumulation of ROS. Excessive ROS not only damages mtDNA and affects the synthesis of key mitochondrial proteins in the OXPHOS process 25, but also cause the impairment of mitochondrial electron transport chain and ATP production. Moreover, they activate the mitochondrial-mediated apoptosis pathway and lead to cell death 26,27. Under pathological conditions, multiple mitophagy pathways are activated and uncontrolled apoptosis and autophagy occur simultaneously 7,28, all of which disrupt cell homeostasis and its normal function. Notably, the pathological alterations mentioned above can interact with each other and form a vicious cycle, which causes various diseases. For example, imbalances in mitochondrial dynamics, calcium dysregulation and genetic defects have been found to be involved in neurodegenerative and metabolic diseases such as Alzheimer's disease, Parkinson's disease and diabetes 29,30,31.

## MITOCHONDRIAL DYSFUNCTION IN OCULAR SURFACE DISEASES

### Dry Eye

Dry Eye (DE), a chronic ocular surface disease affecting approximately 350 million people in China, is characterized with tear film instability and ocular surface inflammation, which might be accompanied with symptoms of ocular discomfort and/or visual dysfunction due to tissue damages and neurological abnormalities 32. With the intensive studies on the pathogenesis of DE, the role of dysregulated oxidative stress homeostasis due to an imbalance between oxidative stress and antioxidants has been explored by many researchers 33.

#### Corneal epithelial cells

**Figure 2:**
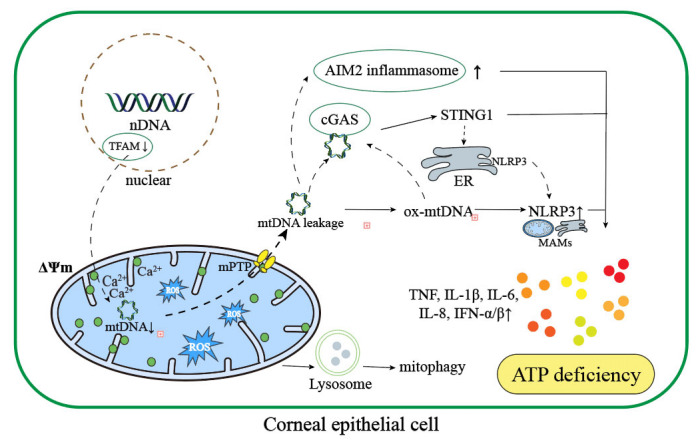
FIGURE 2: The diagram of pathological changes in corneal epithelial cells in DE eyes. The corneal epithelial cells in DE eyes undergo similar pathological changes compared to other cells when mitochondrial dysfunction occurs: an increased level of intracellular oxidative stress, dysregulation of calcium homeostasis, loss of mitochondrial quality control and the occurrence of abnormal mitochondrial dynamics such as mitochondrial autophagy. Several pathways such as cGAS-STING1 pathway and TFAM-AIM2 inflammasome pathway are activated with the destruction of mtDNA. Calcium influx due to the loss of ΔΨm promotes mitophagy and mPTP opening, which causes an increased level of mtDNA leakage into the cytoplasm and abnormally activates the expression and translocation of STING1 via cGAS. NLRP3 activated by ox-mtDNA is translocated and combined to MAMs. All these alterations contribute to an increased cytoplasmic level of pro-inflammatory cytokines such as TNF-α and IL-1ß and the damage of corneal epithelial cells. (MAMs: Mitochondria-associated membranes; ΔΨm: mitochondrial membrane potential; mtDNA: mitochondrial DNA; ox-mtDNA: oxidized mitochondrial DNA; nDNA: nuclear DNA; mPTP: mitochondrial permeability transition pore).

Mitochondrial dysfunction and ROS accumulation in the corneal epithelial cells has been found to be involved in the onset and deterioration of DE (**Figure 2**). When tear osmolarity increases, mtDNA is oxidized and damaged under oxidative stress and binds to NLRP3, the key regulator of ocular surface inflammation 34. Moreover, mtDNA that is released into the cytoplasm through mPTP under stress condition activates the cyclic GMP-AMP synthase (cGAS)-interferon gene stimulator (STING) pathway, aggregating downstream inflammatory responses 35. Recently, it has been reported that a reduced level of mitochondrial transcription factor A (TFAM), a packaging protein of mtDNA that is critical in maintaining mtDNA stability 36,37,38, could impair the function of mitochondria and promote ocular surface inflammation via activating the production of absent in melanoma 2 (AIM2) inflammasome 39.

When mitochondrial dysfunction occurs, it is also commonly seen that the intracellular calcium homeostasis is disturbed because of increased permeability of MAMs, causing an overload of Ca^2+^ in the cytoplasm. The loss of calcium homeostasis results in depolarization of mitochondria and further reduction of ATP production, which impairs corneal epithelial healing in DE eyes 40,41.

#### Lacrimal epithelial cells and meibomian gland epithelial cells

Apart from corneal epithelial cells, mitochondrial dysfunction is also found in lacrimal epithelial cells and meibomian gland epithelial cells. Aging is the independent risk factor for DE 42. The areas of mitochondrial inner membrane area significantly decrease in the lacrimal gland acinar epithelial cells in old rats 43, indicating reduced antioxidant capacity. PINK1/Parkin-mediated mitophagy pathway was found to be involved in the lacrimal gland epithelial cells while aging 44. These studies revealed the pathophysiological mechanism of mitochondrial dysfunction in the damages of lacrimal gland epithelial cell and decreased tear production in old people.

It has been reported that the expression of phosphorylated AMP-activated protein kinase (AMPK) is downregulated in meibomian gland epithelial cells in DE eyes, which might contribute to exacerbated inflammatory responses in meibomian glands and acinar atrophy 45. However, the pathophysiological mechanisms of mitochondrial dysfunction in the damage of meibomian gland epithelial cells and the development of meibomian gland dysfunction have not been fully clarified and require further investigation.

#### Corneal nerve 

Inflammatory infiltration in corneal subbasal nerve plexus and dropout of nerve endings is very common in DE patients 46,47,48. Mitochondrial dysfunction disrupts lipid metabolism in Schwann cells and leads to impaired myelin sheath function and accumulation of toxic lipid intermediates, ultimately resulting in axonal degeneration and neuropathy 49,50. The loss of corneal nerve and corneal hypoesthesia not only impairs the homeostasis of the ocular surface and corneal epithelial regeneration, but also causes reduced tear production and consequent tear hyperosmolarity 51,52,53. The inflammation in local tissue leads to further mitochondrial damages and ROS production, forming a vicious cycle that makes DE progressively worse 54,55.

#### Secondary DE 

It has to be noted that mitochondrial dysfunction is also involved in DE secondary to metabolic diseases, such as diabetes. Hyperglycemia was found to induce severe mitochondrial bioenergetic deficit in lacrimal gland epithelial cells and corneal nerve axons by disrupting the electron transport chain and interfering OXPHOS, leading to widespread oxidative stress, ROS accumulation and cell damage 56,57,58. All these pathological alterations lead to insufficient tear production. Even in young diabetic rats, the expression of nuclear factor erythroid 2-related factor (Nrf2) and HO-1, the stress-responsive enzyme and regulator that reduces oxidative stress and inflammation while maintaining mitochondrial homeostasis, were significantly decreased in the lacrimal glands 59, which supports the clinical findings of early DE onset in diabetic patients 57.

#### Abnormal mitochondrial dynamics

According to the specific needs of cells, mitochondria are in a dynamic balance of fission and fusion. Mitochondrial fission may be a prerequisite for mitophagy 60. Abnormal mitochondrial dynamics lead to mitochondrial fragmentation and eventually mitochondrial dysfunction. However, the mechanism of abnormal mitochondrial dynamics in the pathogenesis of DE has not been fully elucidated. Peng and coauthors reported that adenosine monophosphate-activated protein kinase/mitochondrial fission factor (AMPK/MFF) pathway is involved in the development of DE by positively regulating mitochondrial fission and mitophagy, and the inhibition of key proteins in AMPK/MFF pathway might have therapeutic potentials, which provides new insights in the investigations on the pathogenesis of DE and the development of novel treatment.

### Fuchs endothelial cell dystrophy

Corneal endothelial cells (CEnCs) are responsible for maintaining a relatively dehydration status of cornea, which is dependent on the function of the Na^+^/K^+^-ATPase pump on cell membrane 61. Compared to other cells in cornea, a higher density of mitochondria was identified in CEnCs to ensure the production of a larger amount of ATP 62.

Fuchs endothelial cell dystrophy (FECD), which is characterized by slow degeneration of CEnCs as well as deposition of guttae in both eyes, usually requires keratoplasty to restore visual function because human CEnCs are virtually non-renewable after birth. The pathological characteristics of CEnCs in FECD eyes include oxidative-antioxidant imbalance, stress-induced senescence, mtDNA damage, persistent unfolded protein response 63,64, loss of calcium homeostasis 65 and ER stress 66,67, leading to ROS accumulation, mitochondrial dysfunction and apoptosis of CEnCs 68, as evidenced by the studies in which the damage of mtDNA and nuclear DNA as well as abnormal mitochondrial fission were found in the CEnCs of FECD patients 67. These findings indicate that impaired capacity of mitochondrial respiratory chain plays a crucial role in the pathogenesis of FECD 10. Moreover, recent studies revealed that in patients with late-onset FECD, point mutation of mtDNA A3243G causes CEnC polymorphism and the formation of guttae, while A10398G allele can protect mitochondria from oxidative stress 69,70. Nevertheless, the genotype and phenotype of FECD has not been fully elucidated according to IC3D classification 71. The relationship between mtDNA mutation and the phenotype of FECD merits further investigation.

Abnormal mitochondrial dynamics, especially mitochondrial fission and mitophagy, is also involved in the pathogenesis of FECD, as evidenced by abnormally swollen mitochondria in autophagosomal vacuoles seen in CEnCs under transmission electron microscopy 72. It has been recently reported that the activation of PTEN-induced putative kinase 1 (PINK1)/Parkin pathway is involved in the abnormal mitochondrial mitophagy of FECD, in which the expression of PINK1 on the outer mitochondrial membrane increases and triggers the translocation of Parkin into mitochondria, leading to the ubiquitination of mitochondrial surface proteins and promoting mitophagy 70,73,74.

It is also notable that ER-mitochondrial crosstalk is quite important during stress response 70. ER stress induced by clindamycin in human CEnCs leads to the damage of OXPHOS genes and a reduction of mitochondrial complexes, confirming the role of crosstalk between ER stress and mitochondrial dysfunction in the apoptosis of CEnCs 75.

### Other ocular surface diseases

Being similar with other tissues in diabetic patients, the cornea undergoes a high level of inflammation and oxidative stress 76. As “unified mechanism theory” 77 illustrates, hyperglycemia blocks the mitochondrial electron transfer chain and produces a large amount of ROS 56. Persistent hyperglycemia exacerbates these pathological features and promotes recurrent inflammations. Mitochondrial dysfunction induced by hyperglycemia contributes to the development of diabetic keratopathy, in which the damage of corneal epithelium, CEnCs and nerve plexus was identified 78. Metabolic stress and oxidative damages to cornea nerves and axons are one of the characteristics of diabetic keratopathy. Although NADH and FADH₂ generated by glucose trigger ATP production via OXPHOS in axons under normal condition 79,80,81, this process is impaired under hyperglycemic condition and causes reduced ATP generation and ROS accumulation, which ultimately results in mitochondrial failure and forms a vicious cycle 50,82,83. Mitochondrial dysfunction induced by hyperglycemia contributes to the development of diabetic keratopathy, in which the damage of corneal epithelium, CEnCs and nerve plexus was identified 78. Nevertheless, insulin is beneficial to maintain mitochondrial homeostasis in CEnCs including promoting mitochondrial polarization and respiration and preventing the occurrence of mitochondrial fission under mild stress 84. Moreover, insulin-like growth factor binding protein-3 (IGFBP-3), which plays an anti-inflammatory role in response to oxidative stress, has been reported to restore mitochondrial function of corneal epithelial cells and CEnCs in hyperosmotic models, and inhibit the production and enrichment of mitochondrial inflammatory metabolites in these cells 85.

Keratoconus, a progressive keratectasia disease, has been shown to have inflammation involved in its pathogenesis and present an increased level of mtDNA damage 86,87, which might partially be regulated by TFAM 88,89. Mitochondrial function deficiency and/or decreased capabilities of ROS clearance partially due to lacking antioxidant enzymes are found in keratoconus corneas 89,90, which are likely to cause an elevated level of cellular stress in cornea stroma and lead to collagen degradation and ultimately progressive corneal thinning 20. Nevertheless, the pathogenesis of keratoconus is complicated and the molecular mechanism of mitochondrial dysfunction involved has not been clearly elucidated yet.

## PERSPECTIVE

An increasing number of studies have been published in the recent decade targeting the pathways and mechanisms towards mitochondrial dysfunction, which are helpful to elucidate its role in the pathogenesis of diseases and provide a theoretical basis for novel strategies of mitochondrial therapy. Various novel treatments have been developed targeting the key molecules or proteins involved in mitochondrial dysfunction.

Currently, the treatment of DE is still challenging due to its complicated pathogenesis. However, precision medicines targeting mitochondrial dysfunction may have a better effect than traditional therapies. The Szeto-Schiller peptide (SS-peptide), the mitochondria-penetrating peptide (MPP) and liposomes have been reported to be used as antioxidant delivery systems towards mitochondria 49,91,92. Recently, a novel hierarchical action liposome nanosystem (PHP-DPS@INS) has been reported in the treatment of DE, which could target mitochondria when loading SS-31 peptide and/or insulin and present an antioxidant and anti-inflammatory effect. The dual action of anti-inflammatory and antioxidant breaks the vicious cycle of inflammation in DE eyes 93. In addition, the efficacy of IGFBP-3 is confirmed to restore mitochondrial function in a hyperosmotic model simulating the pathological environment of DE and alleviate DE-related symptoms 85,94. Analogs mimicking the function of TFAM also have potential in the treatment for secondary DE 38,39. Moreover, N-acetyl-L-cysteine and calcitriol are confirmed to be effective to suppress the formation of NLRP3 inflammasome 55,95.

The loss of calcium homeostasis in CEnCs is a main characteristic of FECD. CDGSH iron-sulfur domain-containing protein 2 (CISD2), a protein maintaining calcium homeostasis, has been proved to assist the restoration of human CEnCs *in vitro*, which might have potential for future medication development 40. Additionally, it has been found that under oxidative stress, CEnCs were capable of reacquiring extruded mitochondria through tunneling nanotubes and extracellular vesicles-mediated intercellular transfer 96. Based on this finding, mitochondrial transfer from donor mesenchymal stem cells might have potential to protect corneal epithelial cells from oxidative stress-induced mitochondrial damage, which exploits a new research direction for stem cell therapy 74,97,98.

It should be noted that mitochondrial therapies in the treatment of ocular surface diseases are just at the beginning, being far away from bench to bedside. The efficacy and safety of mitochondrial therapies require further investigation.

## CONCLUSION

The ocular surface is easily been affected by various environmental stress because of its anatomical location. Under pathological stress conditions, mitochondrial dysfunction has been identified in corneal epithelial cells, CEnCs, corneal stromal cells and corneal nerve plexus, and causes various ocular surface diseases. More investigations are necessary to reveal the underlying molecular mechanisms, which is beneficial to unravel novel potential therapeutic targets for mitochondrial therapies.

## CONFLICT OF INTEREST

The authors declare no conflict of interests.
